# Virtual monoenergetic images and post-processing algorithms effectively reduce CT artifacts from intracranial aneurysm treatment

**DOI:** 10.1038/s41598-020-63574-8

**Published:** 2020-04-20

**Authors:** David Zopfs, Simon Lennartz, Lenhard Pennig, Andreas Glauner, Nuran Abdullayev, Johannes Bremm, Nils Große Hokamp, Thorsten Persigehl, Christoph Kabbasch, Jan Borggrefe, Kai Roman Laukamp

**Affiliations:** 1 Institute for Diagnostic and Interventional Radiology, Faculty of Medicine and University Hospital Cologne, University of Cologne, Cologne, Germany; 2 Else Kröner Forschungskolleg Clonal Evolution in Cancer, University Hospital Cologne, Weyertal 115b, 50931 Cologne, Germany; 3 University Hospitals Cleveland Medical Center, Department of Radiology, Cleveland, OH USA; 4 Case Western Reserve University, Department of Radiology, Cleveland, OH USA

**Keywords:** Brain imaging, Computed tomography, Neurology

## Abstract

To evaluate artifact reduction by virtual monoenergetic images (VMI) and metal artifact reduction algorithms (MAR) as well as the combination of both approaches (VMI_MAR_) compared to conventional CT images (CI) as standard of reference. In this retrospective study, 35 patients were included who underwent spectral-detector CT (SDCT) with additional MAR-reconstructions due to artifacts from coils or clips. CI, VMI, MAR and VMI_MAR_ (range: 100–200 keV, 10 keV-increment) were reconstructed. Region-of-interest based objective analysis was performed by assessing mean and standard deviation of attenuation (HU) in hypo- and hyperdense artifacts from coils and clips. Visually, extent of artifact reduction and diagnostic assessment were rated. Compared to CI, VMI ≥ 100 keV, MAR and VMI_MAR_ between 100–200 keV increased attenuation in hypoattenuating artifacts (CI/VMI_200keV_/MAR/VMI_MAR200keV_, HU: −77.6 ± 81.1/−65.1 ± 103.2/−36.9 ± 27.7/−21.1 ± 26.7) and decreased attenuation in hyperattenuating artifacts (HU: 47.4 ± 32.3/42.1 ± 50.2/29.5 ± 18.9/20.8 ± 25.8). However, differences were only significant for MAR in hypodense and VMI_MAR_ in hypo- and hyperdense artifacts (p < 0.05). Visually, hypo- and hyperdense artifacts were significantly reduced compared to CI by VMI_≥140/100keV_, MAR and VMI_MAR≥100keV_. Diagnostic assessment of surrounding brain tissue was significantly improved in VMI_≥100keV_, MAR and VMI_MAR≥100keV_. The combination of VMI and MAR facilitates a significant reduction of artifacts adjacent to intracranial coils and clips. Hence, if available, these techniques should be combined for optimal reduction of artifacts following intracranial aneurysm treatment.

## Introduction

Intracranial aneurysms are a common vascular disorder with a prevalence of approximately 4%, which can be expected to increase due to an ageing population^1,2^. In case of rupture they are associated with a mortality risk of up to 50% [1]. Ruptured as well as unruptured aneurysms are commonly treated with surgical clipping or endovascular coiling^3,4^. Postoperative imaging is pivotal to detect postoperative complications and ensure therapy success for which computed tomography (CT) is frequently applied besides digital subtraction angiography and magnetic resonance imaging^5^. However, evaluation of brain parenchyma adjacent to clips and coils is often impaired by metal artifacts. Artifacts commonly appear as hypo- and hyperdense areas surrounding the metal material. These kind of artifacts result from a combination of beam-hardening caused by absorption of low energetic photons^6,7^, photon starvation resulting from complete absorption of all photons^7,8^, and scatter artifacts that can occur due to greater attenuation differences, e.g. soft tissue and dense metal material^9^. Combined, these effects can result in strong interferences that may impair depiction and interpretation of surrounding brain tissue. Therefore, evaluation of post-interventional complications, such as tissue damage, haemorrhage, infarction and oedema may be limited^10^. Further, any future imaging of the head which might be unconnected to the initial aneurysm treatment, e.g. after accidental trauma or in the event of a stroke, will negatively affect interpretation of CT images due to artifacts from coils and clips.

Altering tube current and voltage and/or applying narrow collimation can reduce metal artifacts, but mostly also increases radiation exposure^11^. Recently, postprocessing iterative metal artifact reduction algorithms (MAR) became available and showed reliable artifact reduction without impact on total applied radiation dose^12,13^. MAR have been reported to yield effective artifact reduction in patients with larger orthopaedic implants^12–14^, and smaller implants, such as dental or intracranial implants^15–17^. Further, MAR have shown artifact reduction in phantom models with coils and clips^10,16,18–20^. Recent studies suggested that combined application of MAR and virtual monoenergetic images (VMI) derived from dual-layer spectral-detector CT (SDCT) may be more effective than individual application of these techniques in different settings^17,21^. However, value of VMI in combination with MAR has not been investigated in post-interventional scans of patients who underwent intracranial aneurysm treatment. Hence, the purpose of this study was to investigate the potential of SDCT derived VMI and MAR reconstructions as a single approach as well as their combination (VMI_MAR_) for metal artifact reduction in patients who underwent intracranial coiling or clipping.

## Results

Our included 35 patients comprised 25 women and 10 men with a mean age of 56.8 ± 13.1 years, ranging from 32–84 years. Twenty-nine were treated in an acute setting after endovascular coiling (n = 15) or surgical clipping (n = 14) due to subarachnoid (n = 21) or intracerebral (n = 8) haemorrhage. Six patients received treatment of aneurysms electively; five of them were coiled and one of them was clipped. Mean time interval between intracranial aneurysm treatment and imaging was 9.8 ± 7.8 days.

### Objective assessment

**Figure 1:**
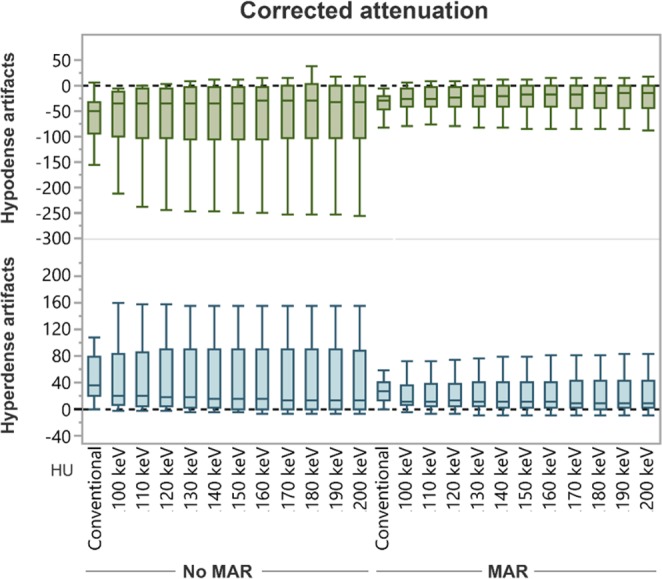
Box-plot diagram displaying corrected attenuation values within hypo- and hyperdense artifacts adjacent to coiling and clipping material in conventional CT images (conventional), virtual monoenergetic images (VMI, 100–200 keV), metal artifact reduction algorithms (MAR) and their combination. Compared to conventional images, corrected attenuation within hypo- and hyperdense artifacts significantly increased and decreased, respectively, in VMI at higher keV. However, the differences were not statistically significant. MAR significantly increased corrected attenuation in hypodense artifacts. In hyperdense artifacts, MAR decreased corrected attenuation, but the difference was not significant. Combination of VMI and MAR at all keV levels significantly increased corrected attenuation in hypodense and decreased corrected attenuation in hyperdense artifacts.

The Shapiro-Wilk test did not show normal distribution for values in all assessed groups (p < 0.05). Compared to CI, corrected attenuation in VMI at higher keV within hypo- and hyperdense artifacts significantly increased (CI/VMI_200keV_: −77.6 ± 81.1/−65.1 ± 103.2 HU) and decreased (47.4 ± 32.3/42.1 ± 50.2 HU), respectively (Fig. 1). However, the differences were not statistically significant. MAR significantly increased corrected attenuation in hypodense artifacts (−36.9 ± 27.7 HU, Table 1). In hyperdense artifacts, MAR decreased corrected attenuation (29.5 ± 18.9 HU), but the difference was not significant (Table 1). Combination of VMI and MAR at all keV levels significantly increased corrected attenuation in hypodense (VMI_MAR200keV_: −21.1 ± 26.7 HU) and decreased corrected attenuation in hyperdense artifacts (20.8 ± 25.8 HU, Fig. 1, Table 1). More detailed results on differences between reconstructions can be found in supplementary Tables 1 & 2.

**Table 1:** Objective assessment of artifact reduction and surrounding tissue.

	Corrected attenuation		Corrected image noise	
	Hypodense artifact	Hyperdense artifact	Hypodense artifact	Hyperdense artifact
CI	(−)77.6 ± 81.1	47.4 ± 32.3	35.9 ± 35.4	17.5 ± 13.8
**VMI**				
*100 keV*	(−)71.3 ± 92.9	44.4 ± 44.7	32.1 ± 38.5	18.7 ± 18.5
*140 keV*	(−)66.9 ± 99.8	42.9 ± 48.5	30.4 ± 41.2	18.4 ± 19.2
*200 keV*	(−)65.1 ± 103.2	42.1 ± 50.2	30.3 ± 42.3	18.4 ± 20.0
MAR	**(−)36.9** ± **27.7**	29.5 ± 18.9	**12.2** ± **14.1**	**7.5** ± **5.4**
**VMI-MAR**				
*100 keV*	**(−)23.9** ± **21.8**	**22.5** ± **21.3**	**9.8** ± **8.0**	**7.2** ± **7.4**
*140 keV*	**(−)21.9** ± **24.7**	**21.0** ± **24.6**	**9.0** ± **7.8**	**7.2** ± **8.1**
*200 keV*	**(−)21.1** ± **26.7**	**20.8** ± **25.8**	**8.9** ± **8.0**	**7.2** ± **8.1**
**p-values**				
CI vs. VMI 100–200 keV	p > 0.05	p > 0.05	p > 0.05	p > 0.05
CI vs. MAR	p = 0.047	p = 0.34	p = 0.004	p = 0.058
CI vs. VMI-MAR 100–200 keV	p < 0.05	p < 0.05	p < 0.05	n/a

CI - conventional images; VMI - virtual monoenergetic images; MAR - Artifact reduction algorithms; VMI-MAR - combination of MAR and VMI; significant changes in HU-values compared to CI are marked in bold (p < 0.05), n/a - not available.

**Figure 2:**
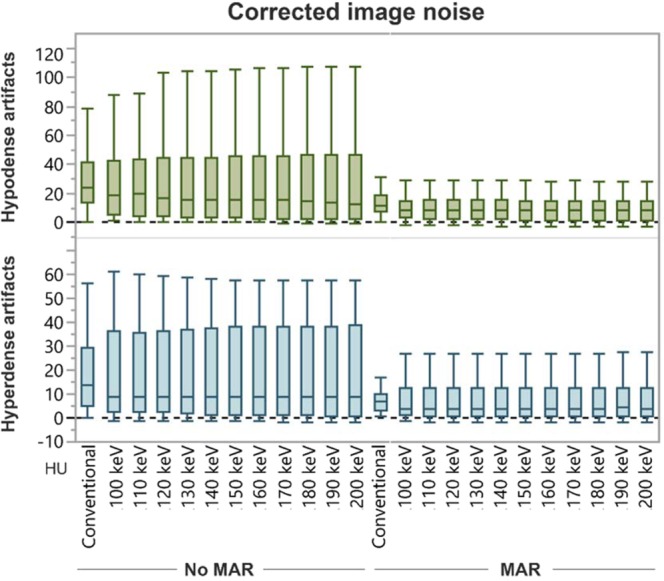
Box-plot diagram displaying corrected image noise values within hypo- and hyperdense artifacts adjacent to coils and clipping material in conventional CT images (conventional), virtual monoenergetic images (VMI, 100–200 keV), metal artifact reduction algorithms (MAR) and their combination. Compared to conventional images, corrected image noise slightly decreased in hypodense artifacts in VMI ≥ 100 keV and slightly increased in hyperdense artifacts in VMI at all keV-values; however, differences were not significant. MAR alone and in combination with VMI at all keV-levels significantly decreased image noise compared to CI.

Compared to CI, corrected image noise slightly decreased in hypodense artifacts in VMI ≥ 100 keV and slightly increased in hyperdense artifacts in VMI at all keV-values; however, differences were not significant. MAR alone and in combination with VMI at all keV-levels significantly decreased corrected image noise compared to CI (CI/VMI_MAR200keV_, hypodense: 35.9 ± 35.4/8.9 ± 8.0 HU; hyperdense: 17.5 ± 13.8/7.2 ± 8.1 HU, Fig. 2, Table 1).

### Visual assessment

**Figure 3:**
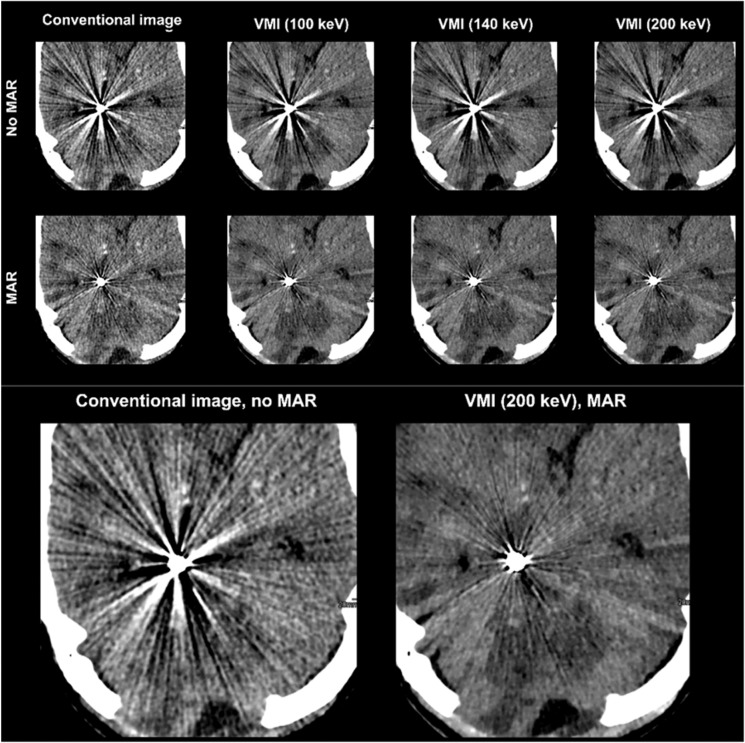
Combination of VMI and MAR reduce even strongest artifacts and relevantly improve assessment of adjacent brain tissue. Conventional CT images (conventional), virtual monoenergetic images (VMI, 100–200 keV), metal artifact reduction algorithms (MAR) and their combination.

**Figure 4:**
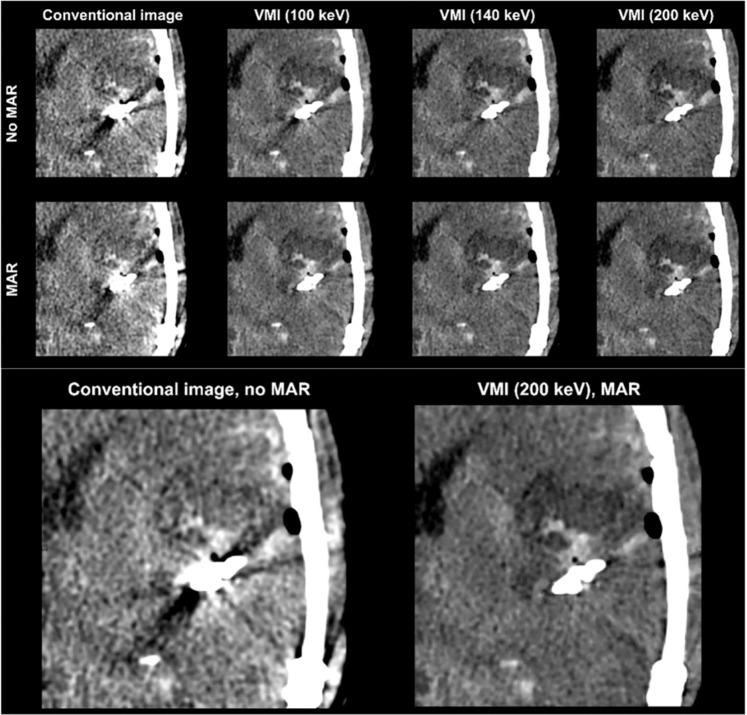
Combination of VMI and MAR completely reduces artifacts and reveals surrounding postsurgical bleeding and brain defect. Conventional CT images (conventional), virtual monoenergetic images (VMI, 100–200 keV), metal artifact reduction algorithms (MAR) and their combination.

**Figure 5:**
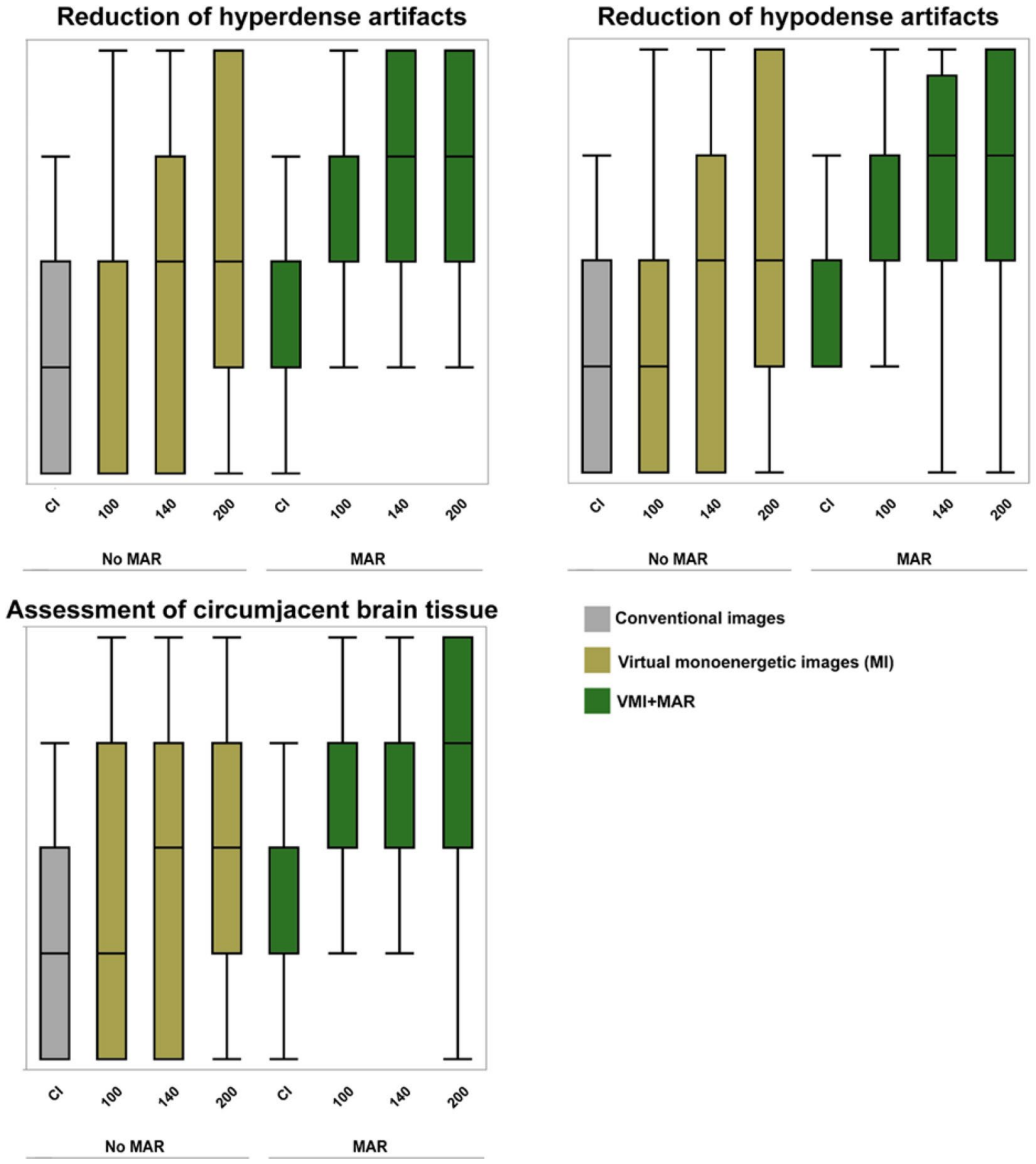
Box-plot diagrams displaying visual artifact reduction and diagnostic assessment of circumjacent brain tissue in conventional CT images (CI), virtual monoenergetic images (VMI, 100–200 keV), metal artifact reduction algorithms (MAR) and their combination.

Compared to CI, VMI ≥ 140 keV, MAR and combination of VMI and MAR significantly reduced hypodense artifacts (CI/VMI_200keV_/MAR/VMI_MAR200keV_, 2(1–3)/3(1–5)/3(2–4)/4 (2–5), Figs. 3–5, Table 2). Hyperdense artifacts were significantly lower in VMI at 100 keV, MAR and combination of VMI and MAR (CI/VMI_200keV_/MAR/VMI_MAR200keV_, 2(1–4)/3(1–5)/3(2–4)/4(3–5), Figs. 3–5, Table 2). Diagnostic assessment of brain tissue adjacent to coils or clips was significantly improved in VMI ≥ 100 keV, MAR and combination of VMI and MAR (VMI_MAR_). However, best results were generated for the combination of VMI and MAR (CI/VMI_200keV_/MAR/VMI_MAR200keV_, 2(1–3)/3(1–5)/2(1–4)/4(2–5), Table 2). Patient examples are given in Figs. 3 and 4.

**Table 2:** Subjective assessment.

	Artifact extent		Diagnostic assessment of surrounding brain tissue	New artifacts	
	Hypodense	Hyperdense		Local	Cerebellum
CI	2 (1–3)	2 (1–4)	2 (1–3)	n/a	n/a
**VMI**					
*100 keV*	2 (1–4)	**3 (1–4)**	**2 (1–4)**	3 (3–3)	2 (2–2)
*140 keV*	**3 (1–4)**	**3 (1–5)**	**3 (1–5)**	3 (3–3)	2 (2–2)
*200 keV*	**3 (1–5)**	**3 (1–5)**	**3 (1–5)**	3 (3–3)	2 (2–2)
MAR	**3 (2–4)**	**3 (2–4)**	**2 (1–4)**	2.5 (1–3)	1 (1–2)
**VMI-MAR**					
*100 keV*	**3 (2–5)**	**3 (2–5)**	**3 (2–5)**	3 (2–3)	1 (1–2)
*140 keV*	**4 (2–5)**	**4 (2–5)**	**4 (2–5)**	3 (2–3)	1 (1–2)
*200 keV*	**4 (2–5)**	**4 (3–5)**	**4 (2–5)**	3 (2–3)	1 (1–2)
ICC	0.68	0.65	0.69	0.57	
**p-values**					
CI vs. VMI 100 keV	p > 0.05	p = 0.025	p < 0.001	n/a	n/a
CI vs. VMI 140 keV	p < 0.001	p < 0.001	p < 0.001	n/a	n/a
CI vs. VMI 200 keV	p < 0.001	p < 0.001	p < 0.001	n/a	n/a
CI vs. MAR	p < 0.001	p < 0.001	p < 0.001	n/a	n/a
CI vs. VMI-MAR 100–200 keV	p < 0.001	p < 0.001	p < 0.001	n/a	n/a

New artifacts - Introduction of overcorrection or new artifacts compared to conventional images; CI - conventional images, VMI - virtual monoenergetic images, MAR - Artifact reduction algorithms, VMI-MAR - combination of MAR and VMI; significant changes in scores compared to CI are marked in bold (p < 0.05); ICC - Intraclass correlation coefficient; Data is reported as median and 10/90-percentile.

Overcorrection of artifacts due to coils and clips was not noticed for VMI. Overcorrection was reported for MAR in 24% of patients and for VMI_MAR_ in 40–42% of patients (100 keV/140 keV/200 keV: 40%/42%/40% of patients, respectively). Furthermore, no new artifacts were noted for VMI alone. However, for MAR additional artifacts were reported in 24% of patients and for VMI_MAR_ in 6–8% of patients (100 keV/ 140 keV/200 keV: 8%/ 6%/ 6% of patients). New artifacts not resulting from aneurysm repair mostly occurred in the posterior fossa at MAR in 71% of patients and using VMI_MAR_ in 71% of patients (100 keV/140 keV/200 keV: 71%/71%/71% of patients). In VMI alone, no new artifacts in other brain areas or posterior fossa were reported.

Mean optimal keV values for diagnostic assessment were 179.6 ± 32.4 keV (range, 100–200 keV) for VMI and 169.9 ± 32.5 keV (range, 100–200 keV) for VMI_MAR_. Optimal keV values were significantly lower for combination of MAR and VMI compared to VMI alone (p = 0.008).

Overall interrater agreement was good (intraclass correlation coefficient: 0.66, Table 2).

## Discussion

This study assessed VMI and MAR as well as their combination (VMI_MAR_) for artifact reduction in unenhanced dual-energy SDCT of the head after surgical clipping or endovascular coiling of intracranial aneurysms. Combined VMI_MAR_ at higher keV yielded best results and allowed for an effective artifact reduction as well as improvement of diagnostic assessment of circumjacent brain tissue. VMI at higher keV levels could only reduce artifacts in the subjective reading but failed to reduce artifacts significantly in the objective assessment. In line, MAR reduced visual appearance of artifacts and improved diagnostic assessment while only allowing for significant reduction of hypodense artifacts in the objective assessment.

In line with our results, recent studies reported that combination of VMI and MAR proved to be most effective compared to each method on its own for artifacts from different devices^15,16,22^. A recent phantom study performed with a dual-source dual-energy CT scanner revealed that artifacts caused by coils and clips could also be most effectively reduced using the combination of virtual monoenergetic imaging and dedicated metal artifact reduction algorithms^23^. Still, to our best knowledge, this is the first study that evaluates the combination of VMI and MAR for artifact reduction in patients after endovascular coiling or surgical clipping of aneurysm. Further, this combination has not been investigated on a dual-layer SDCT so far. Regarding earlier studies, there has been controversy regarding either method on its own providing sufficient artifact reduction. Particularly for stronger artifacts, VMI and MAR as a standalone approach have been reported to yield suboptimal artifact reduction capacity^15,16,18,20,24^. Concordantly, our study demonstrated that VMI and MAR reduced artifacts in the visual assessment to some extent yet fail to significantly reduce artifacts as determined by quantitative measurements. Previous studies that investigated reduction of artifacts caused by intracranial aneurysm repairment facilitated by either VMI or MAR as a single approach mostly found significant artifact reduction, still complete or sufficient artifact reduction was not achieved^3,10,19,24,25^. Our results and those from the aforementioned dual-source CT phantom study of Winklhofer *et al*.^23^ found a dedicated advantage using both approaches VMI and MAR in combination.

Artifact reduction by VMI and MAR postprocessing is available from all major vendors^12,26^. Effectiveness of artifact reduction differs regarding the different types of implants and locations within the human body. For several implants either VMI and MAR as standalone approaches have been successfully used to reduce artifacts, e.g. total hip replacements, spinal fixation hardware, contrast media, and dental implants^12,13,22–24,27,28^. This aspect is highly relevant as combination of both approaches is only available in dual-energy CT systems which allow for row data-based reconstruction of virtual monoenergetic images (VMI), but clinically used are mainly conventional single-energy CT systems, thereby MAR as a standalone post processing approach has the advantage of a higher availability also in conventional CT systems^12,13^. However, at least in the objective assessment MAR failed to reduce hyperdense artifacts. Nevertheless, we consider the sole application of MAR as a reasonable attempt in non-dual-energy CT systems to raise image quality and diagnostic assessment.

As we wanted to focus our analysis on clinically relevant artifacts, we did not include patients with rather subtle artifacts. Still, earlier studies have suggested that artifact reduction of VMI and/or MAR in these patients is less challenging and complete reduction of artifacts is achieved relatively often^15,17,23^.

In our study, artifact reduction improved at higher keV levels in VMI and VMI_MAR_, especially, the subjective readers found a distinctive advantage and recommended high keV levels in most patients. Mean optimal keV values for diagnostic assessment were significantly lower in combination of VMI with MAR (~170 keV) compared to VMI (~180 keV) alone. Due to their physical properties higher keV VMI reduce brain tissue contrast^26,29^, the combination of VMI and MAR enables to receive optimal artifact reduction at lower keV values, where loss of tissue contrast is lower^14,22,26,29^. To achieve optimal results, we therefore recommend using the combination VMI and MAR between 140 to 200 keV. The optimal keV values differs between patients and should therefore be adjusted individually.

In our analysis, as a limitation, overcorrection of initial artifacts has been reported for MAR and in combination with VMI which is concordant with findings from earlier studies^10,15,16^. Still, the overcorrection appeared in areas in which initial artifacts appeared in CI and thereby did not additionally impair image quality or diagnostic assessment. Only in very few cases new artifacts that additionally impaired diagnostic assessment of brain tissue were noted, confirming existing literature^10,16^. Moreover, in areas of the brain not initially affected by artifacts, new artifacts were reported which happened most commonly in the posterior fossa. Thus, in clinical routine using SDCT with VMI_MAR_ corrected images for improved evaluation of adjacent brain tissue after aneurysm coiling or clipping in case of artifacts at the posterior fossa additional uncorrected conventional reconstructions might be helpful. As especially MAR imposes the relevant risk of introduction of new artifacts we recommend applying it only when clinically warranted. Although new artifacts were mostly not negatively impacting diagnostic assessment in the area of interest around the coil and clipping material. Further, we recommend to only use MAR reconstructions or their combination with VMI in areas where artifacts are present. In other areas, CI should stay standard of care. Especially in the posterior fossa where MAR often introduced new artifacts.

For clinical interpretation of our results the following additional limitations need to be considered. First, the approach how to measure artifacts needs to be discussed. In artifact reduction studies, the standard approach is a ROI based measurement of mean and standard deviation of attenuation and also offers advantages as relatively easy applicability and is therefore mostly used in artifact reduction studies^22,30,31^. However, also more complex methods have been applied using dedicated artifact quantification algorithms^16,32^. Second, general changes in attenuation and image noise appear in VMI of different keV levels due to their differing physical properties^26^. To only detect real artifact reduction opposed to general changes of attenuation and image noise, we applied an intra-individual comparison between artifact impaired tissue and correspondent non-artifact impaired reference tissue resulting in the corrected image noise and corrected attenuation^15^. To address possible limitations of the objective assessment we included a complementary subjective evaluation. Third, we did not aim for complete blinding for reconstructions as images (CI, VMI, VMI_MAR_) were distinguishable by their appearances. We aimed to encourage readers to appreciate subtle differences between reconstructions next to a qualitative rating of images; therefore, readers were always presented with a full image set of one patient at a time. Another limitation concerns our selection process, as only patients with clinically relevant artifacts and decision by the responsible radiologist to perform additional MAR were included. This way rather patients with stronger and diagnostic impairing artifacts were selected, so that rather subtle artifacts were left out.

## Conclusion

In artifacts resulting from endovascular or classic neurosurgical cerebral aneurysm treatment, combination of VMI and MAR yielded best results for artifact reduction and should therefore be used in a combined approach if available. Without this option, MAR can still be used as an attempt to facilitate diagnostic assessment of brain tissue circumjacent to aneurysm coiling or clipping.

## Methods

This study was approved by the local institutional review board (Ethics committee of the Faculty of Medicine from the University of Cologne) and is in accordance with the ethical regulations of the 1964 Helsinki declaration as well as later amendments. Due to the retrospective study design the local institutional review board waived necessity for informed consent. Criteria for inclusion were as follows:

(i) unenhanced SDCT examinations of the head in patients that received aneurysm treatment in form of coils or clips causing diagnostic assessment impairing artifacts;
(ii) imaging performed within a maximal time interval of 30 days after treatment;
(iii) availability of MAR reconstructions in addition to conventional and spectral image reconstructions.

MAR reconstructions were clinically only applied on behalf of the responsible radiologist when strong artifacts were present hampering diagnostic assessment. This resulted in the inclusion of 35 patients and SDCT examinations between June 2018 and May 2019. In five patients repetitive imaging occurred. These examinations were excluded from further analysis.

### Imaging protocol

All examinations were conducted on a clinical spectral-detector CT (SDCT, IQon, Philips Healthcare). Head-first supine position was used in all patients. Scanning was performed for clinical reasons only; no scan was conducted explicitly for the study. Unenhanced examinations of the head were performed using the following scan parameters: collimation 64 × 0.625 mm, rotation time 0.5 s, pitch 0.390, matrix 512 × 512, computed tomography dose index 44.7 mGy. Tube voltage was set to 120 kVp and tube current was set to 260 mAs. Mean dose length product (DLP) was 847.1 ± 175.2 mGy*cm.

Conventional CT images (iDose4, level 3; Philips Healthcare; referred to as CI), MAR (O-MAR; Philips Healthcare), VMI (Spectral B, level 3, Philips Healthcare, range: 100–200 keV, increment of 10 keV), and the combination of MAR and VMI (Spectral B, level 3 and O-MAR, Philips Healthcare, range: 100–200 keV, increment of 10 keV) were reconstructed. Slice thickness was set to 0.9 mm.

### Objective analysis

Image assessment was conducted using regions of interest (ROI) with a consistent size of 50 mm^2^. First, ROI were placed in CI and then copied to MAR, VMI and VMI_MAR_ using the vendor’s proprietary image viewer (IntelliSpace Portal v9, Philips Healthcare). ROI were placed in hypo- and hyperdense artifacts as well as corresponding artifact free reference tissue, e.g. when a hyperdense artifact impaired white matter parenchyma, artifact free contralateral white matter parenchyma was selected as a reference tissue; this method was used individually for each artifact. Mean and standard deviation within each ROI were recorded. Standard deviation in artifact impaired brain tissue was considered representative for artifact burden^33^.

In addition, corrected attenuation for hypo- and hyperdense artifacts were calculated as the difference between attenuation in artifact impaired and non-affected artifact free reference tissue^15^. This method accounts for general changes in attenuation along altering keV values of VMI to minimize any bias and detect real artifact reduction^34^. Additionally, corrected image noise^21^ was calculated as the difference between image noise in artifact impaired and artifact free reference tissue to correct for general lower image noise in higher keV VMI^34^.

### Visual analysis

Four radiologists, with six, five, three and two years of experience in neuroradiology rated extent of hypo- and hyperdense artifacts on 5-point Likert-scales independently (Table 3). Further, diagnostic assessment of brain tissue surrounding coils and clips was evaluated on 5-point Likert-scale. For evaluation of diagnostic assessment readers were asked to consider detection/exclusion of interventional/postsurgical complications, such as brain tissue damage, haemorrhage, infarction and oedema (Table 3). Moreover, overcorrection or new artifacts adjacent to coils or clips were reported on a tertiary scale (Table 3). Overcorrection was defined as artifacts occurring in the same location as the initial artifacts not impairing image assessment compared to CI. New artifacts were defined as artifacts in the same or other locations as the initial artifacts that additionally impaired image assessment compared to CI. Furthermore, new artifacts in other parts of the brain without association with the cerebral aneurysm repair were rated on a binary scale (Table 3). Readers also reported optimal keV value for overall diagnostic assessment considering extent of artifact, possible overcorrection and/or new artifacts, as well as brain tissue contrast which has been reported to decline in high keV VMI.

**Table 3:** Visual analysis.

Extent of hypo- and hyperdense artifacts	(5) artifacts are absent/almost absent;
	(4) minor artifacts;
	(3) moderate artifacts;
	(2) pronounced artifacts;
	(1) massive artifacts
Diagnostic assessment of brain tissue surrounding endovascular coils and surgical clips	(5) full diagnostic quality as there were no artifacts or almost no artifacts;
	(4) marginally affected diagnostic quality by minor streaks;
	(3) hampered diagnostic quality by moderate artifacts;
	(2) restricted diagnostic quality by strong artifacts;
	(1) insufficient diagnostic quality
Presence of overcorrection or new artifacts compared to CI in the region of interest adjacent to endovascular coils and surgical clips	(3) no overcorrection or new artifacts;
	(2) overcorrection, new opposite artifact in the location of initial artifacts without additional impairment of diagnostic assessment compared to CI; (1) new artifacts in the same or different location of initial artifacts with additional impairment of diagnostic assessment compared to CI
Presence of new artifacts compared to CI unrelated to the region of interest	(2) no new artifacts;
Monoenergetic level with best diagnostic assessment	(1) new artifacts

CI – Conventional images.

Readers were blinded to the results of the objective analysis. Due to visually different appearances of the available reconstructions, full blinding appeared not feasible for consistent evaluation. Therefore, readers were provided with a complete set of images for each patient, including CI, MAR, VMI, and VMI_MAR_. VMI and VMI_MAR_. KeV values for VMI and VMI_MAR_ were 100, 140, and 200 keV. Larger increments than in the objective assessment were applied to allow for detection of relevant changes in image assessment that might otherwise be obscured by rating images appearing too similar due to smaller keV value increment. Image parameters were as follows: slice thickness 0,9 mm, axial plane, initial window level: 35 and window width: 70. Readers were explicitly free to adjust window settings manually, as both artifacts and different VMI keV-levels have a distinctive influence on optimal window settings^26^.

### Statistical analysis

Statistical analyses were conducted using JMP Software (V14, SAS Institute, Cary, USA). Quantitative results are presented as mean ± standard deviation and qualitative results are displayed as median and 10/90 percentile. Shapiro-Wilk test was applied to test for normal distribution. Wilcoxon-test with Steel adjustment for multiple comparisons was used to test for any significant difference. Statistical significance was defined as p < 0.05. Interreader agreement was assessed using the intraclass correlation coefficient (ICC). Interpretation was conducted as proposed earlier: agreement being poor <0.40; fair 0.40–0.59; good 0.60–0.75; and excellent 0.75–1.0^35^.

## Supplementary information

Supplementary Table 1–3.

## Data Availability

The data that support the findings of this study are available from the corresponding author upon reasonable request.
