# Supplementary material for: Virtual monoenergetic images and post-processing algorithms effectively reduce CT artifacts from intracranial aneurysm treatment
Source: Sci Rep. 2020 Apr 20;10:6629. doi: 10.1038/s41598-020-63574-8 (PMC7170914; doi:10.1038/s41598-020-63574-8)
Supplement: Supplementary file 1 — Supplementary Table 1–3. [file 41598_2020_63574_MOESM1_ESM.docx]

**Virtual monoenergetic images and post-processing algorithms effectively reduce CT artifacts from intracranial aneurysm treatment**

**Authors:**

David Zopfs^1^, Simon Lennartz^1,2^, Lenhard Pennig^1^, Andreas Glauner^1^, Nuran Abdullayev^1^, Johannes Bremm^1^, Nils Große Hokamp^1^, Thorsten Persigehl^1^, Christoph Kabbasch^1^, Jan Borggrefe^1^, Kai Roman Laukamp^1,3,4,^*

**Affiliations:**

^1^Institute for Diagnostic and Interventional Radiology, Faculty of Medicine and University Hospital Cologne, University of Cologne, Cologne, Germany

^2^Else Kröner Forschungskolleg Clonal Evolution in Cancer, University Hospital Cologne, Weyertal 115b, 50931, Cologne, Germany

^3^University Hospitals Cleveland Medical Center, Department of Radiology, Cleveland, OH, USA

^4^Case Western Reserve University, Department of Radiology, Cleveland, OH, USA

*Corresponding author

**Corresponding author:**

Kai Roman Laukamp

Institute for Diagnostic and Interventional Radiology, University Hospital Cologne, Kerpener Straße 62, 50937 Cologne, Germany

Telephone: +49 221 478-82035

Telefax: +49 221 478-82384

kai.laukamp@uk-koeln.de

| **Supplementary Table 1 - Detailed objective measurements** | | | | | | | | |
| --- | --- | --- | --- | --- | --- | --- | --- | --- |
|  | Attenuation | | | | Image noise | | | |
|  | Hypodense artifact | Reference tissue | Hyperdense artifact | Reference tissue | Hypodense artifact | Reference tissue | Hyperdense artifact | Reference tissue |
| CI | (-)45.3±80.9 | 32.3±3.5 | 80.3±32.2 | 32.8±2.9 | 42.4±35.9 | 6.6±1.6 | 24.4±13.8 | 6.9±1.6 |
| VMI |  |  |  |  |  |  |  |  |
| *70 keV* | (-)50.1±90.0 | 31.2±3.2 | 80.5±38.6 | 32.4±2.6 | 43.3±35.5 | 4.8±1.3 | 25.2±16.4 | 4.9±1.3 |
| *80 keV* | (-)43.5±86.7 | 30.8±2.8 | 77.4±41.2 | 31.0±2.3 | 40.0±36.1 | 4.7±1.3 | 24.2±17.1 | 4.7±1.2 |
| *90 keV* | (-)43.6±89.2 | 30.0±2.5 | 75.4±43.6 | 30.2±2.2 | 38.0±37.4 | 4.6±1.2 | 23.6±17.8 | 4.6±1.2 |
| *100 keV* | (-)41.8±92.4 | 29.4±2.5 | 74.0±45.4 | 29.6±2.3 | 36.7±38.6 | 4.6±1.2 | 23.3±18.3 | 4.6±1.2 |
| *140 keV* | (-)38.5±99.1 | 28.4±3.3 | 71.4±49.3 | 28.5±2.2 | 34.9±41.2 | 4.5±1.2 | 22.9±19.4 | 4.5±1.2 |
| *200 keV* | (-)37.0±102.5 | 28.0±2.4 | 70.3±51.2 | 28.1±2.4 | 34.8±42.3 | 4.5±1.2 | 22.8±19.8 | 4.4±1.2 |
| MAR | (-)2.5±25.3 | 34.4±15.4 | 61.7±19.3 | 31.6±5.0 | 20.4±10.6 | 8.2±7.6 | 14.4±5.8 | 6.8±1.8 |
| VMI-MAR |  |  |  |  |  |  |  |  |
| *70 keV* | 2.6±25.2 | 31.5±3.5 | 57.1±20.2 | 32.1±2.7 | 18.8±13.4 | 5.6±3.5 | 13.5±6.9 | 4.8±1.1 |
| *80 keV* | 3.8±21.4 | 30.3±3.1 | 54.7±19.5 | 30.8±2.4 | 16.6±10.5 | 5.6±4.3 | 13.1±8.1 | 4.7±1.1 |
| *90 keV* | 4.7±20.7 | 29.5±2.8 | 53.2±20.4 | 30.0±2.2 | 15.3±9.1 | 5.1±2.2 | 12.6±8.8 | 4.6±1.0 |
| *100 keV* | 5.2±21.0 | 28.9±2.8 | 51.9±20.8 | 29.4±2.3 | 14.6±8.4 | 5.0±2.0 | 12.6±8.8 | 4.6±1.0 |
| *140 keV* | 6.1±23.5 | 27.9±2.7 | 49.5±25.0 | 28.5±2.1 | 13.6±8.0 | 5.3±4.5 | 12.1±8.2 | 4.5±1.0 |
| *200 keV* | 6.0±25.4 | 27.5±2.8 | 48.8±26.4 | 28.0±2.2 | 13.4±8.1 | 5.2±3.9 | 12.7±9.8 | 4.5±1.0 |
| *CI - conventional images; VMI - virtual monoenergetic images; MAR - Artifact reduction algorithms; VMI-MAR - combination of MAR and VMI* | | | | | | | | |

| **Supplementary Table 2 - Additional p-values of the objective results** | | | |
| --- | --- | --- | --- |
|  |  | Corrected attenuation | |
|  |  | Hypodense artifact | Hyperdense artifact |
| p-values |  |  |  |
| VMI vs. VMI-MAR | *100 keV* | **p<0.05** | p=0.123 |
|  | *110 keV* | **p<0.05** | p=0.173 |
|  | *120 keV* | **p<0.05** | p=0.201 |
|  | *130 keV* | **p<0.05** | p=0.228 |
|  | *140 keV* | p=0.066 | p=0.197 |
|  | *150 keV* | p=0.084 | p=0.197 |
|  | *160 keV* | p=0.108 | p=0.247 |
|  | *170 keV* | p=0.131 | p=0.267 |
|  | *180 keV* | p=0.297 | p=0.242 |
|  | *190 keV* | p=0.151 | p=0.237 |
|  | *200 keV* | p=0.155 | p=0.252 |
| VMI-MAR vs. MAR | *100 - 200 keV* | p>0.05 | p>0.05 |
| VMI vs. MAR | *100 - 200 keV* | p>0.05 | p>0.05 |
| *VMI - virtual monoenergetic images; MAR - Artifact reduction algorithms; VMI-MAR - combination of MAR and VMI, significant changes in HU-values are marked in bold* | | | |
|  |  |  |  |
|  |  |  |  |
|  |  |  |  |

| **Supplementary Table 3 - Additional p-values of the subjective results** | | | | |
| --- | --- | --- | --- | --- |
|  |  | Artifact extent | | Diagnostic assessment of surrounding brain tissue |
|  |  | Hypodense artifact | Hyperdense artifact |  |
| p-values |  |  |  |  |
| VMI vs. VMI-MAR | *100 - 200 keV* | **p<0.001** | **p<0.001** | **p<0.001** |
| VMI-MAR vs. MAR | *100 - 200 keV* | **p<0.001** | **p<0.001** | **p<0.001** |
| VMI vs. MAR | *100 keV* | **p<0.001** | p>0.05 | p>0.05 |
|  | *140 keV* | p>0.05 | p>0.05 | p>0.05 |
|  | *200 keV* | p>0.05 | p>0.05 | **p<0.001** |
| *VMI - virtual monoenergetic images; MAR - Artifact reduction algorithms; VMI-MAR - combination of MAR and VMI, significant changes are marked in bold* | | | | |
|  |  |  |  |  |
|  |  |  |  |  |
